# Tuberculous peritonitis after conservative treatment for acute perforated appendicitis: a case report

**DOI:** 10.1186/s40792-024-01928-4

**Published:** 2024-05-21

**Authors:** Satoru Tanoue, Yuki Ohya, Osamu Nakahara, Hirotaka Maruyama, Aritome Norifumi, Takeshi Morinaga, Tsugio Eto, Akira Tsuji, Shintaro Hayashida, Hidekatsu Shibata, Hironori Hayashi, Mitsuhiro Inoue, Kazumi Kuriwaki, Masayoshi Iizaka, Yukihiro Inomata

**Affiliations:** 1 Department of Surgery, Kumamoto Rosai Hospital, 1670 Takehara-Machi, Yatsushiro, Kumamoto 866-8533 Japan; 2 Department of Surgery, Taragi Municipal Hospital, 4210 Taragi, Kuma District, Kumamoto, 868-0501 Japan; 3 Department of Respiratory Medicine, Kumamoto Rosai Hospital, 1670 Takehara-Machi, Yatsushiro, Kumamoto 866-8533 Japan; 4 Department of Diagnostic Pathology, Kumamoto Rosai Hospital, 1670 Takehara-Machi, Yatsushiro, Kumamoto 866-8533 Japan

**Keywords:** Tuberculous peritonitis, Acute perforated appendicitis, Abscess

## Abstract

**Background:**

Interval appendectomy is widely recommended for patients with abscesses due to perforated appendicitis. A concomitant malignancy-related problem was reported after conservative treatment of acute appendicitis with abscess, but perforated appendicitis-associated tuberculous peritonitis was never reported.

**Case presentation:**

A 67-year-old male patient with a laryngeal cancer history presented to our hospital with an acute appendicitis-associated ileal abscess. He was scheduled for an interval appendectomy after conservative treatment. Fortunately, the symptoms subsided, and the patient was discharged for a later scheduled appendectomy. However, after 3 months, he was readmitted to our hospital with fever and abdominal pain, and emergency surgery was performed, which was suspected to be peritonitis. Intraoperative results revealed numerous white nodules in the abdominal cavity. The condition was diagnosed as tuberculous peritonitis based on macroscopic results, later pathological findings, and positive T-SPOT.TB. The antituberculosis medications were effective, and the patient recovered and was discharged from the hospital 8 days thereafter.

**Conclusion:**

Patients, particularly those immunocompromised, may develop tuberculous peritonitis after conservative treatment for acute perforated appendicitis.

## Background

Interval appendectomy is widely accepted for treating acute perforating appendicitis with localized abscess [1, 2]. Conversely, interval appendectomy in such a condition requires caution because of the high frequency of malignancy [2–4]. However, the occurrence of tuberculous peritonitis was not reported. The lung is one of the most prevalently infected organs in patients with tuberculosis, and extrapulmonary tuberculosis constitutes 15–20% of all tuberculosis cases [2, 5, 6]. Tuberculous appendicitis is rare in the gastrointestinal tract [7, 8]. Herein, we report a case of tuberculous peritonitis 3 months after nonoperative treatment of a patient with acute appendicitis and abscess.

## Case presentation

**Fig. 1:**
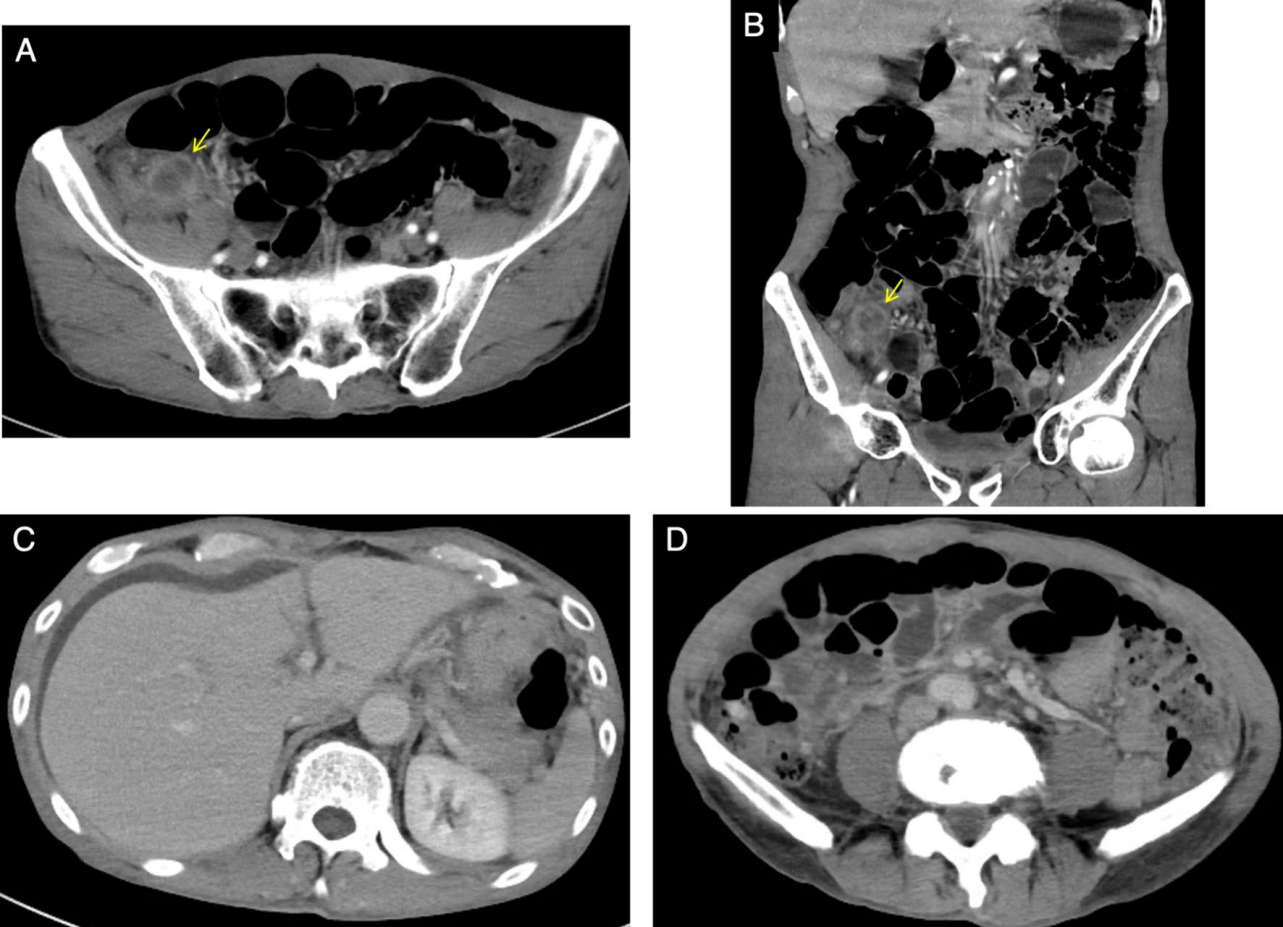
Abdominal enhanced CT findings. **A**, **B** CT showing appendicular abscess. Yellow arrows show appendicular abscess. **C** CT showing peritoneal thickening and ascites. **D** CT showing an ileal abscess. *CT* computed tomography

**Fig. 2:**
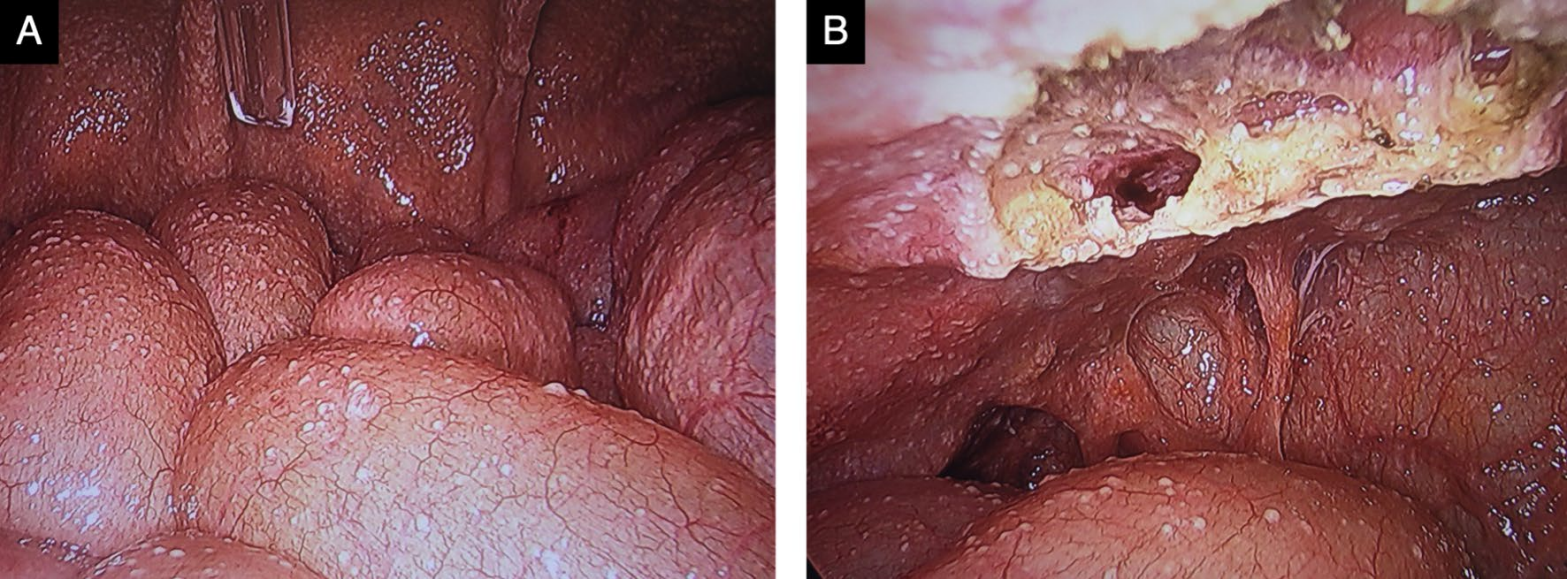
Intraoperative findings. **A**, **B** Numerous white nodules on the peritoneum, intestine, and mesentery

**Fig. 3:**
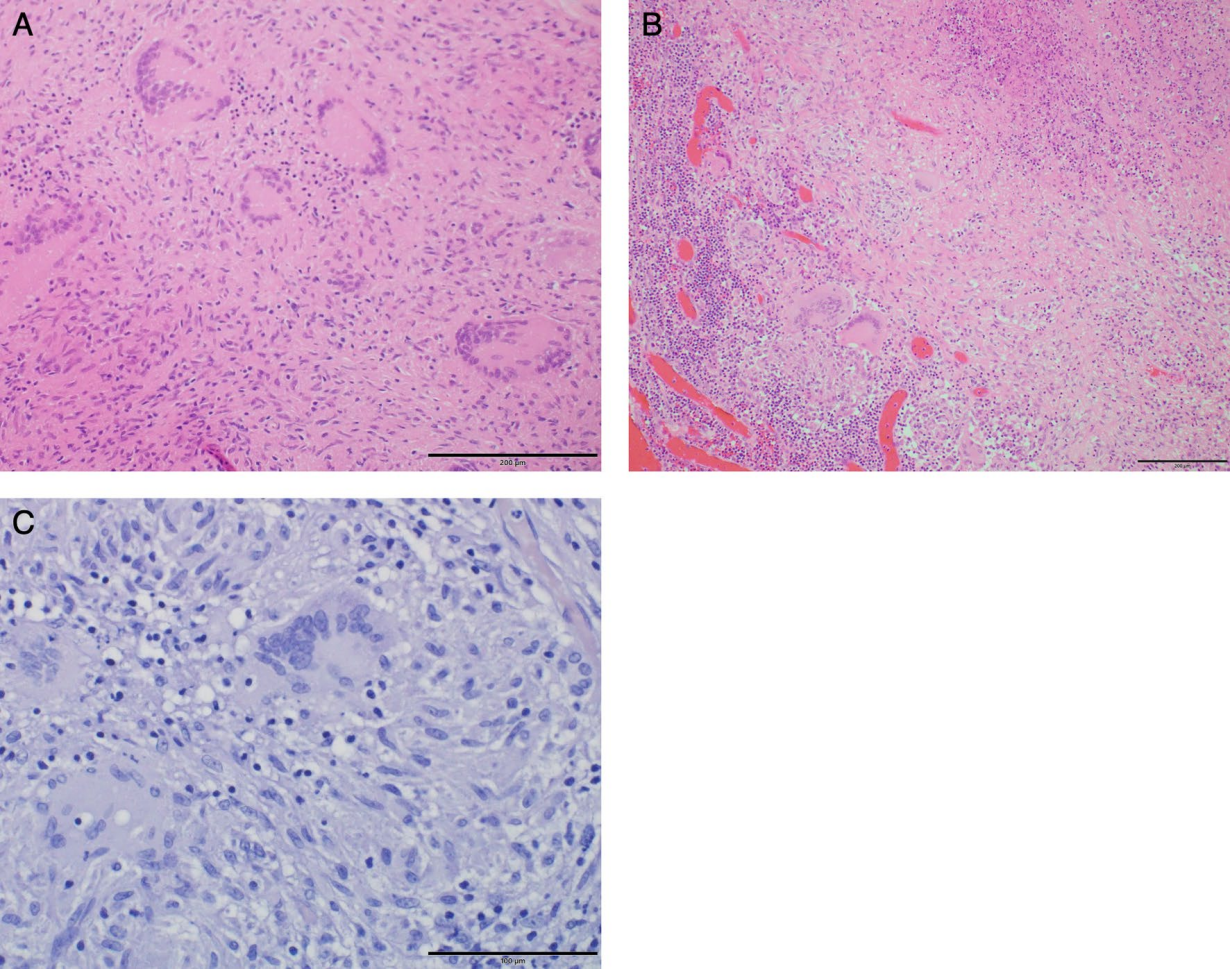
Pathological findings. The resected white nodule (**A**) and appendix (**B**) showing epithelial granuloma with necrosis and multinucleated giant cells and no malignant results or caseous necrosis. **C** The Ziehl–Neelsen stain was negative in the resected appendix. Scale bar: 200 μm (**A**, **B**) and 100 μm (**C**)

**Fig. 4:**
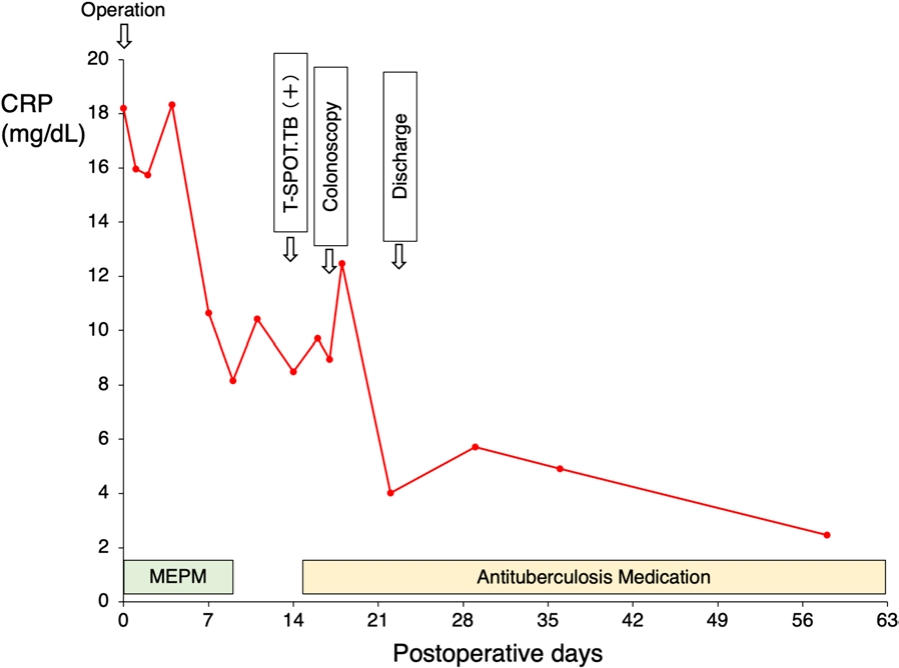
Perioperative changes in CRP. The patient’s CRP promptly decreased after antituberculosis treatment initiation, and the patient was discharged from the hospital. *CRP* C-reactive protein

A 67-year-old male patient had diarrhea and fever for the past 10 days and presented to our hospital with a chief complaint of right lower abdominal pain. Contrast-enhanced computed tomography (CT) revealed acute appendicitis perforation with abscess (Fig. 1A, B). He had a history of surgery and radiation chemotherapy for laryngeal cancer 6 months before the episode. He had no history of tuberculosis infection or any lung lesions suggesting the past tuberculosis on CT. The patient was scheduled for conservative treatment followed by interval appendectomy. At that time, we did not suspect appendicular tuberculosis and just considered the abscess to be due to perforation of the ordinary acute appendicitis. The patient was treated conservatively with meropenem without administration of antituberculosis medicine and was discharged after 13 days when his symptoms subsided. He has been followed up as an outpatient since then. However, after 3 months, he was readmitted to our hospital with a fever and abdominal pain. In this case, the patient was diagnosed with peritonitis not localized by CT, and emergency laparoscopic surgery was performed (Fig. 1C, D). No obvious inflammation or perforation of the appendix was observed. The appendix was firmly adhesed to the cecum. Gastrointestinal perforation was not observed and an obvious cause of inflammation could not be identified. Laparoscopic surgery was converted to laparotomy for appendicectomy and peritoneal drainage. Intraoperative results revealed numerous white nodules in the abdominal cavity, which were considered peritoneal dissemination of the malignancy or tuberculous peritonitis (Fig. 2). Intraoperative cytology revealed no malignant cells. The ascites culture taken during the operation was negative. However, the pathological results of the resected nodule and appendix demonstrated epithelial granuloma with necrosis and multinucleated giant cells, indicating the possibility of tuberculous peritonitis originating from the appendix (Fig. 3). However, tuberculous peritonitis could not be definitively diagnosed based on the pathology alone. Thus, T-SPOT.TB was performed 11 days postoperatively. It was positive, and antituberculosis treatment was initiated 14 days postoperatively. The treatment was a 6-month regimen including isoniazid, rifampin, pyrazinamide and ethambutol for 2 months, followed by isoniazid plus rifampin for 4 months. A colonoscopy was attempted for the possibility of intestinal tuberculosis 17 days postoperatively, but it could not be passed into the ileum. The patient’s symptoms improved quickly after the initiation of antituberculosis medications, and he was discharged from the hospital 22 days postoperatively (Fig. 4).

## Discussion

Acute appendicitis is a prevalent disease. Interval appendectomy for perforated appendicitis is relatively prominent [1–4], but no studies reported tuberculous peritonitis following the treatment of perforated appendicitis. However, a study reported two cases that indicated tuberculous peritonitis a few months after intestinal perforation in patients with malignancy [9]. The duration from perforation to the onset of tuberculous peritonitis in that report was similar to that in our case. In our case, the appendiceal abscess was relieved conservatively without administration of antituberculosis medicines. Therefore, the pathogenesis of our patients is theoretically inferred to be similar to that of the patients in that report. Our patient had most likely a perforation of usual appendicitis, not appendicular tuberculosis. Anyway, pathological diagnosis of the resected appendix is important because it may help the diagnosis of appendiceal tuberculosis.

T-SPOT.TB is a type of ELISpot assay used for tuberculosis diagnosis, which belongs to the group of interferon-gamma release assays. In this case, postoperatively performed positive T-SPOT.TB confirmed the possibility of tuberculosis [10, 11]. According to the World Gastroenterology Organisation Global Guidelines for digestive tract tuberculosis [12], interferon-gamma assay such as T-SPOT is a highly sensitive test for the diagnosis of tuberculous peritonitis. Therefore, T-SPOT.TB has been useful in tuberculous peritonitis diagnosis. However, according to the guideline, we should have also considered measuring the adenosine deaminase of ascites and performing PCR in ascitic fluid. Tuberculous peritonitis was considered to be certain clinically because of the antituberculosis medication was effective. Awareness of the possibility of tuberculous peritonitis is important in the treatment of patients with acute perforated appendicitis, even in those without a tuberculosis history. Perforated appendicitis can be accompanied by malignancy [2–4]. Therefore, attention should be paid not only to tuberculous peritonitis, but also to malignancy in patients with perforated appendicitis. Acute appendicitis may cover appendiceal tuberculosis or malignancy, and pathological examination of the resected appendix in acute appendicitis is important.

In this case, we did not anticipate tuberculous peritonitis preoperatively. Therefore, we performed the surgery without any prophylaxis for infection. Fortunately, no TB infection was confirmed in the medical staff after the surgery. Since it is difficult to diagnose tuberculous peritonitis preoperatively, it is important to prepare for the possibility of aerosol transmission intraoperatively when tuberculous peritonitis is suspected on intraoperative findings [13].

## Conclusion

The possibility of developing tuberculous peritonitis should be considered, especially in immunocompromised patients, such as those with malignancy, concerning conservative treatment of acute appendicitis with perforation.

## Data Availability

Not applicable.

## Abbreviation

CT: Computed tomography
